# Aligned laminin core-polydioxanone/collagen shell fiber matrices effective for neuritogenesis

**DOI:** 10.1038/s41598-018-23958-3

**Published:** 2018-04-03

**Authors:** Su-Jin Song, Yong Cheol Shin, Sung Eun Kim, Il Keun Kwon, Jong-Ho Lee, Suong-Hyu Hyon, Dong-Wook Han, Bongju Kim

**Affiliations:** 1 Department of Cogno-Mechatronics Engineering, College of Nanoscience & Nanotechnology, Pusan National University, Busan, 46241 Republic of Korea; 2 Research Center for Energy Convergence Technology, Pusan National University, Busan, 46241 Republic of Korea; 3 Department of Dental Materials, School of Dentistry, Kyung Hee University, Seoul, 02447 Republic of Korea; 4 Department of Oral and Maxillofacial Surgery, School of Dentistry, Seoul National University, Seoul, 03080 Republic of Korea; 5 Center for Fiber and Textile Science, Kyoto Institute of Technology, Matsugasaki, Kyoto 606-8585 Japan; 6 Dental Life Science Research Institute, Seoul National University Dental Hospital, Seoul, 03080 Republic of Korea

## Abstract

Neural tissue regeneration is a significant challenge, because severe nerve injury is quite difficult to regenerate spontaneously. Although, many studies have been devoted to promote nerve regeneration, there are still many technical challenges to achieve satisfactory results. In this study, we designed biomimetic matrices composed of aligned laminin core-polydioxanone/collagen shell (Lam-PDO/Col) fibers, which can provide both topographical and biochemical cues for promoting neuritogenesis. The aligned Lam-PDO/Col core-shell fiber matrices were fabricated by magnetic field-assisted electrospinning with the coaxial system, and their potential as biofunctional scaffolds for promoting neuritogenesis was explored. It was demonstrated that the aligned Lam-PDO/Col core-shell fibers were successfully fabricated, and the laminin in the core of fibers was steadily and continuously released from fibers. In addition, the cellular behaviors of hippocampal neuronal cells on the matrices were significantly enhanced. Moreover, the aligned Lam-PDO/Col fiber matrices effectively improved and guided neurite outgrowth as well as the neurogenic differentiation by providing both topographical and biochemical cues through aligned fiber structure and sustained release of laminin. Collectively, it is suggested that the aligned Lam-PDO/Col core-shell fiber matrices are one of the most promising approaches for promoting neuritogenesis and neural tissue regeneration.

## Introduction

Nerve injury is often caused by resections of tumors, traffic accidents or iatrogenic side effects of the surgery^1^. A slight nerve injury can be recovered spontaneously, while severe nerve injury is quite difficult to regenerate spontaneously. Therefore, numerous efforts have been devoted to effectively promote nerve regeneration. However, conventional strategies, such as nerve coaptation or the reconstruction with allografts, xenografts and autografts, have several limitations owing to the limited supply and the risk of immune-rejection or ineffectiveness in severe nerve injury^2,3^. Therefore, much research have focused on the development of artificial scaffolds to enhance the neuronal cell behaviors by a tissue engineering approach. Several techniques, such as gas forming, salt leaching and electrospinning, have been employed for fabricating artificial scaffolds that can mimic the natural extracellular matrix (ECM) strucuture^4–6^. Among these techniques, electrospinning is the technique of using a high electric field generated between a polymer solution in a syringe and a grounded collector^7–10^. Hence, the electrospinning techniques have attracted a significant attention as a simple and effective technique for fabricating tissue engineering scaffolds^11^. Previous studies have demonstrated that various structures of artificial matrices can be fabricated by the electrospinning^12^. In addition, the aligned fibrous matrices can enhance or induce the differentiation of stem cells into specific cell lineages, such as neurogenic, chondrogenic, osteogenic, and myogenic lineages^13–16^. Especially, aligned fibrous matrices are suitable for the regeneration of neuronal cells because the aligned fibers can provide an axon guidance cue to neuronal cells, which is effective to neurogenic differetiation^13,17,18^. It has been documented that the neurite outgrowth can be enhanced by aligned fibrous structure around neuronal cells^19^. Therefore, there have been many efforts to fabricate aligned fibrous scaffolds using various biocompatible and biodegradable polymer such as polylactids, polycaprolactones and poly(lactic-*co*-glycolic acid) by electrospinning for tissue regeneration^20^. Meanwhile, various biochemical cues, such as proteins, peptides, drugs, and growth factors, can be incorporated into fibrous matrices for further enhancing tissue regeneration^21–23^. However, successful delivery and steady stimulation by bioactive molecules during the entire tissue regeneration process remain significant challenges in the rational designing of scaffolds. To address this issue, core-shell fibers have been proposed as one of the most desired strategies for controllably and sustainably delivering the bioactive molecules^24–26^. The release rate of encapsulated biomolecules in the core of fibers can be adjusted by choosing an appropriate shell material, which can leads to the desired release rate of bioactive molecules in a controlled manner. In this study, we designed fibrous matrices composed of aligned laminin core-polydioxanone/collagen shell (Lam-PDO/Col) fibers, which can provide both topographical and biochemical cues for promoting neuritogenesis, and investigated their effects on neuronal cell regeneration.

In general, neural tissue regeneration takes a relatively long time that may take several weeks^27,28^. Therefore, scaffolds for neural tissue regeneration should have long-term biodegradable time to provide a long enough time for nerve regeneration and maturation. PDO is a biocompatible polymer, and has long-term biodegradable time, good flexibility and mechanical properties. In addition, since PDO exhibits superior biocompatibility and low inflammatory response^29,30^, it has been widely used for various biomedical and clinical applications including drug delivery carrier, surgical suture material and tissue engineering scaffold^29,31^. However, its intrinsic hydrophobic property is a major obstacle to its use as a tissue engineering scaffold. Hence, we used PDO in a combination with Col to improve the hydrophilicity of fiber matrices. Col, a main component of ECMs, is a highly hydrophilic material, and plays a crucial role in cell adhesion. According to the previous studies, Col-blended electrospun matrices can improve the cell adhesion and proliferation^32^. Meanwhile, Lam was encapsulated in the core region of aligned fibers to specifically promote neuronal cell growth and neurite outgrowth. Lam is an abundant glycoprotein in basement membrane of nerve tissues, and composed of multidomain “cross-link” shaped structure consisting of three polypeptide chains (α1, β1 and β2)^33^. Lam in the nerve system can not only promote neurite outgrowth, but can also provide a specific axonal guidance^34^. On the other hand, fiber alignment plays a key role in determining cellular behaviors, including cell alignment, migration and differentiation^35,36^. To fabricate aligned nanofibers, particulary designed collectors, such as a parallel electrode collector or fast ratating collector, have been used. In those electrospining systems, the degree of fiber alignment is highly dependent on the rotating speed or the width of gap between parallel electrodes^37–40^. Meanwhile, there have been continuous efforts to achieve highly aligned structure using electrospinning. A magnetic field-assisted electrospinning is one of the most useful techniques for fabricating highly aligned nanofiber matrices. It has been reported that the magnetic field-assisted electrospinning allows fabricating the highly aligned nanofiber structures by supplying electrical and magnetic fields to electrostatical charged polymer nanofibers^39,41,42^. The external magnetic field can be introduced at a collector where the insulating and grounding regions are alternately arranged, and subsequently the charged fibers are uniaxially stacked onto the collector. Herein, we fabricated aligned Lam-PDO/Col core-shell fiber matrices by magnetic field-assisted electrospinning with the coaxial system. In addition, the physicochemical and mechanical properties of aligned Lam-PDO/Col core-shell matrices were characterized, and the degree of fiber alignment was analyzed by the fast Fourier transform (FFT) method^43^. Furthermore, the cellular behaviors of HT-22 mouse hippocampal neuronal cells on the matrices were evaluated to examine their potentials as tissue engineering scaffolds for neuritogenesis and neural tissue regeneration.

## Materials and Methods

### Fabrication of aligned Lam-PDO/Col core-shell matrices

The random PDO, aligned PDO, aligned PDO/Col, and aligned Lam-PDO/Col core-shell matrices were produced by magnetic field-assisted electrospinning (Figure S1). Briefly, random PDO matirces were fabricated with the 160 mg/mL of the PDO resins (Center for Fiber and Textile Science, Kyoto Institute of Technology, Kyoto, Japan) dissolved in 1, 1, 1, 3, 3, 3-hexafluoro-2-propanol (HFIP, Sigma-Aldrich, MO, USA). The as-prepared PDO solution was loaded into a syringe (HSW NORM-JECT, Tuttlingen, Germany) with a 23-gauge spinneret needle, and continuously injected at a flow rate of 0.5 mL/h. A distance between the collector and needle tip was 16 cm, and an applied voltage was adjusted at 13–15 kV. The aligned PDO matrices were prepared by magnetic field-assisted electrospinning at 2000 rpm of collector rotation (Figure S1B)^39,41,42^. A working distance and the flow rate were same with the condition of random PDO matrices. The aligned PDO/Col matrices were fabricated with the 240 and 12 mg/mL of PDO and Col (Darim Tissen, Seoul, Korea) in HFIP, respectively. The aligned Lam-PDO/Col core-shell matrices were fabricated by magnetic field-assisted electrospinning with the coaxial system that could encapsulate Lam in the core region of the electrospun fibers. The PDO (240 mg/mL) and Col (12 mg/mL) were dissoved in HFIP to obtain shell solution, and the Lam (30 μg/mL; Gibco, Invitrogen, Singapore) was dissolved in distilled water to obtain the core solution. The inner and outer needles had a diameter of 25-gauge and 18-gauge, respectively. The shell and core solutions loaded in each syringe were separately supplied by two syringe pumps at flow rates of 1 and 0.3 mL/h, respectively. A voltage of 15 kV was supplied to the shell solution, and the electric fields were simultaneously transferred to the core solution. The fabricated Lam-PDO/Col core-shell fibers were accumulated on a rotating collector (2000 rpm), followed by slow vacuum-drying at room temperature for overnight to eliminate the residual solvent. Afterwards, the prepared fiber matrices were cut into a round shape with 9 mm diameter. Prior to use, all samples were sterilized under ultraviolet (UV) light.

### Characterization of aligned Lam-PDO/Col core-shell matrices

The surface morphologies of the matrices were examined by a field emission scanning electron microscope (FESEM; Hitachi S4700, Tokyo, Japan) at a 5 kV acceleration voltage. Before examination, all matrices were sputter coated with a thin layer of platinum. The degree of fiber alignment was analyzed using FFT method^43–46^. The FFT analysis converts the information of a real domain from its orignial image data to a mathematically defined frequency domain. The FESEM images of fabricated matrices were analyzed using ImageJ software (National Institutes of Health, Bethesda, MD, USA) with an oval profile plug-in (designed by William O’Connnell)^43^, and the results of FFT analysis indicate the degree of fiber alignment. The distribution of FFT frequency was obtained by an oval projection of FFT analysis images, followed by a radial summation of the pixel intensities between 0 and 360° (Figure S2), and presented by plotting integrated pixel intensities. The distributions of FFT frequency were symmetric, which in turn the integrated pixel intensities were presented between 0 and 180°. All FFT analysis results were normalized to compare data sets obtained in each matrices (i.e. random PDO, aligned PDO, aligned PDO/Col and aligned Lam-PDO/Col core-shell matrices) using ORIGIN 8.0^®^ software (OriginLab Corporation, Northampton, MA, USA).

Compositional analysis of the matrices was performed by Fourier transform infrared (FTIR) spectroscopy. The FTIR spectra of fabricated matrices were recorded by a Nicolet 560 FTIR spectroscope (Nicolet Co., Madison, WI, USA) in absorption mode at the wavelength ranging from 500 cm^−1^ to 3500 cm^−1^ with 4.0 cm^−1^ resolution and 16 scans.

The mechanical properties of the matrices were examined by universal testing machine (LRX Plus, Lloyd Instruments Ltd., Fareham, UK) with a 5 kN load cell. Four types of matrices were cut into a rectangular shape with 10 mm in width and 40 mm in length. The tensile strength, elastic modulus and elongation at break of matrices were calculated from the stress-strain curves obtained at a cross-head speed of 10 mm/min.

The surface hydrophicility of the matrices was investigated by measuring the water contact angle. The sessile drop of a distilled water (10 μL) was placed on the matrices, and the contact angles were measured by using OCA10 goniometer (Dataphysics, Filderstadt, Germany).

### *In vitro* laminin release and *in vitro* degradation analysis

To examine the *in vitro* release profile of Lam from Lam-PDO/Col core-shell matrices, the matrices were immersed in 5 mL Dulbecco’s phosphate-buffered saline (DPBS, pH 7.4; Gibco BRL, Rockville, MD, USA), and then incubated at 37 °C in a benchtop shaker (Lab Companion Model SI-600R, Jeio Tech Co., Ltd., Daejeon, Korea) with shaking at 80 rpm for up to 28 days. After incubation, the UV and visible (UV/Vis) absorption at 281 nm was monitored by a UV/Vis spectrophotometer (Evolution™ 220, Thermo Fisher Scientific, Waltham, MA, USA). The concentration of Lam released from matrices was expressed as the percentage of its loading amounts.

The random PDO, aligned PDO, aligned PDO/Col and aligned Lam-PDO/Col core-shell matrices were cut into 10 × 10 mm squares, and their cumulative weight loss (%) was measured. Weighed matrices were immersed in 5 mL DPBS, and then incubated at 37 °C in a benchtop shaker with shaking at 80 rpm for up to 4 weeks. The matrices were retrieved at determined time intervals and rinsed thoroughly with distilled water, followed by slow vacuum-drying at room temperature. The mass loss of matrices was measured, and presented as the percentage of the each weight of matrices at each time point to its initial weight.

### Cell culture conditions and *in vitro* assays for cellular behaviors

The neuronal cell behaviors on aligned Lam-PDO/Col core-shell matrices were investigated using HT-22 hippocampal neuronal cells. HT-22 cell is an established cell line as a hippocampal neuron model that has been extensively used for investigating the neuritogenesis and nerve tissue engineering^47–52^. HT-22 cell line is a sub-line derived from the HT-4 cell line, and immortalized with a SV-40 T antigen^47,49^. HT-22 cell line shows the most similar behavior to undifferentiated neuronal cells, and expresses neuron-specific properties^51,52^. HT-22 cells were routinely cultured in growth media, Dulbecco’s modified Eagle’s medium (DMEM, Welgene, Daegu, Korea) containing 10% fetal bovine serum (Welgene) and 1% antibiotic-antimycotic solution (10,000 units of penicillin, 25 μg/mL of amphotericin B, and 10 mg of streptomycin, Sigma-Aldrich) at 37 °C in a humid incubator with 5% CO_2_^39^.

The initial cell attachment and proliferation were measured by a cell counting kit-8 assay (CCK-8 assay, Dojindo, Kumamoto, Japan) following the manufacturer’s instruction. The HT-22 hippocampal neuronal cells were seeded at 1 × 10^4^ cells/mL on the random PDO, aligned PDO, aligned PDO/Col, and aligned Lam-PDO/Col core-shell fibers matrices, and incubated for 6 h to 7 days. After the each culture period of incubation (6 h for initial cell attachment and 1, 3, 5, and 7 days for proliferation measurement, respectively), the CCK-8 solution was added, and incubated for 2 h at 37 °C in the dark. Subsequently, the absorbance values were measured at 450 nm using an SpectraMax 340 ELISA reader (MolecularDevice Co., Sunnyvale, CA, USA).

The neurogenic differentiation was induced in differentiation medium, neurobasal medium (Gibco) containing 2% B-27 supplement (Gibco), 4% fetal bovine serum and 1% antibiotic-antimycotic solution. The HT-22 cells were seeded at 1 × 10^4^ cells/mL on the random PDO, aligned PDO, aligned PDO/Col, and aligned Lam-PDO/Col core-shell fiber matrices in growth medium. After 5 days of incubation, the culture media were replaced from growth media to differentiation media, and then the HT-22 hippocampal cells were continuously incubated at 37 °C in a humid incubator with 5% CO_2_ for 7 days.

### Immunofluorescence staining analysis

Immunofluorescence staining was implemented at nuclei, filamentous-actins (F-actins) and neurofilaments of HT-22 hippocampal neuronal cells. The cells were incubated for 5 days to observe the morphology of nuclei and F-actins, and they were used after cell culture in differentiation medium for additional 7 days to observe neurofilaments. The neurofilament is a valuable and classical marker for neuronal differentiation, and it has been extensively used as a typical marker for neuronal differentiation and neuritogenesis^53–57^. After incubation in each condition, HT-22 cells were treated with a 3.7% formaldehyde solution (Sigma-Aldrich) for 10 min, and permeabilized in 0.1% Triton X-100 (Sigma-Aldrich) for 5 min, followed by blocking with a 5% bovine serum albumin (BSA, GenDEPOT, Barker, TX, USA) solution for 30 min. Subsequently, the cells were incubated with tetramethyl rhodamine isothiocyanate (TRITC, 1:40 in 1% BSA solution; Molecular Probes, Eugene, OR, USA) for 20 min in the dark at room temperature. The neurofilaments were immunostained with anti-neurofilament heavy polypeptide antibody (Abcam, Cambridge, MA, USA), followed by a secondary goat anti-rabbit IgG heavy and light (H&L) chains (Abcam) conjugated with fluorescein isothiocyanate (FITC). The nuclei were counterstained with a 4′,6-diamidino-2-phenylindole (DAPI, 1 μM, Sigma-Aldrich) solution in DPBS. The fluorescence imaging was conducted using a custom-built two-photon laser fluorescence microscopy, as described elsewhere^58–60^. The average neurite length was calculated from the two-photon excitation fluorescence images. A neurite length was measured as a distance from the end of neurite to the junction between neurite base and cell body^17^. All measurements were carried out using ImageJ software.

### Statistical analysis

All variables were tested in the three independent measurements for each experiment, and repeated two times (the number of samples for each group; n ll6). All presented data are expressed as average ± standard deviation. Prior to statistical analysis, the Levene’s test was carried out to analyze data for the quality of variances. After a one-way analysis of variance (SAS Institute Inc., Cary, NC, USA), statistical group comparisons were conducted by a Bonferroni test. A statistically significance is defined as a value of *p* < 0.05.

## Results and Discussion

### Fiber characteristics of aligned Lam-PDO/Col core-shell matrices

**Figure 1:**
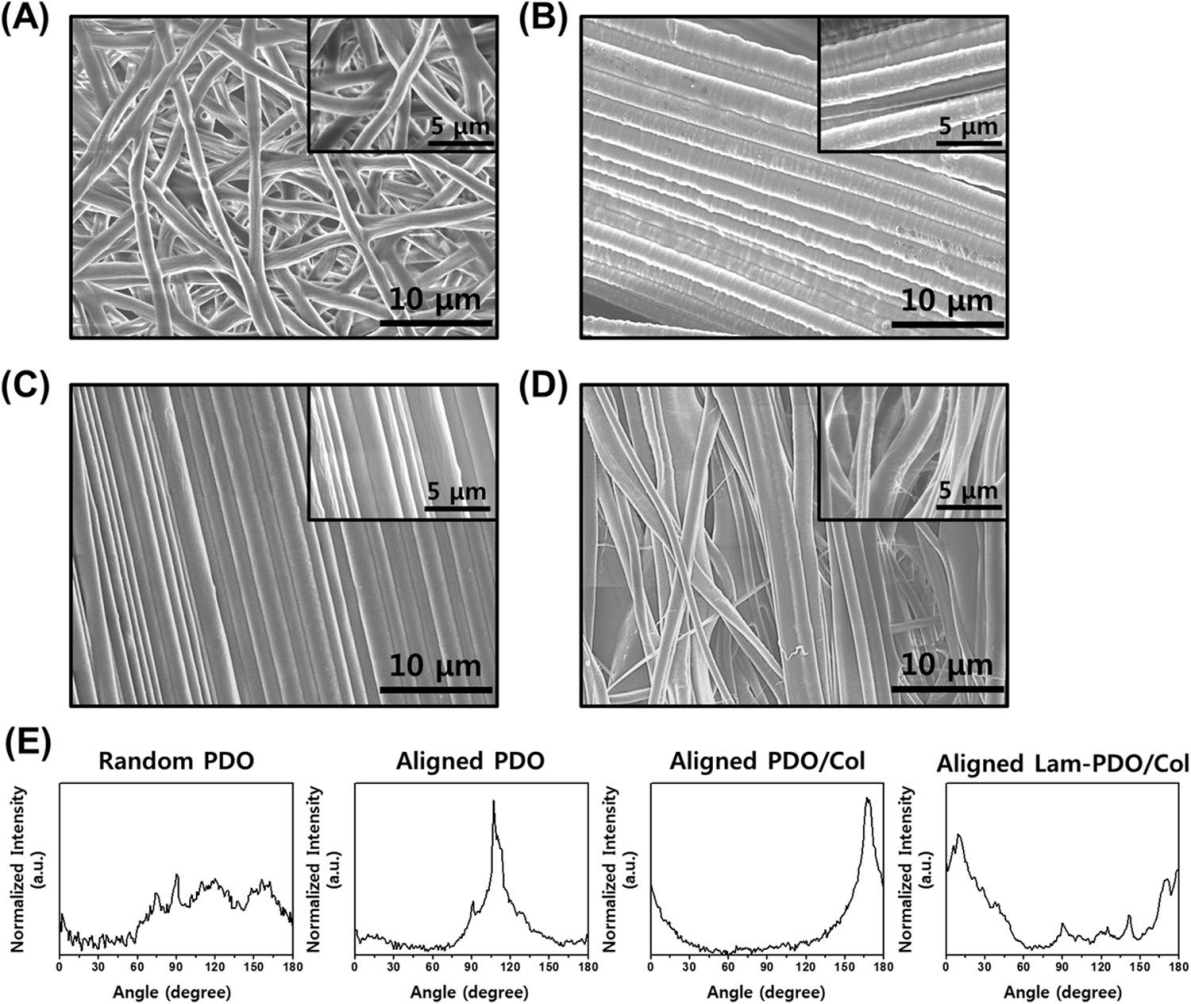
Surface characteristics of the random PDO, aligned PDO, aligned PDO/Col, and aligned Lam-PDO/Col core-shell matrices. Representative FESEM images of (**A**) the random PDO, (**B**) aligned PDO, (**C**) aligned PDO/Col, and (**D**) aligned Lam-PDO/Col core-shell matrices. (**E**) Pixel intensity plots of fiber alignment for the random PDO, aligned PDO, aligned PDO/Col, and aligned Lam-PDO/Col core-shell matrices.

The digital photographs of random PDO, aligned PDO, aligned PDO/Col, and aligned Lam-PDO/Col core-shell matrices were shown in Figure S3A. The alignment of fibers could be readily observed with the naked eye. Figure 1A–D showed the surface morphologies of fiber matrices obtained from FESEM images. The random PDO matrices were found to have a three-dimensional interconnected pore structure that were similar to the natural ECM (Fig. 1A). There were no beads in random PDO fibers, indicating that the random PDO matrices were developed successfully as scaffolds. In the aligned PDO, PDO/Col and Lam-PDO/Col core-shell matrices, it was shown that the electrospun fibers were aligned successfully (Fig. 1B–D). The FFT analysis of an original FESEM image of random fiber matrices produces an output image where pixels are distributed in a symmetrical and circular shape because the frequency in which pixel intensities are distributed in data images is metaphysically uniform in any direction (Figure S2)^43^. On the other hand, FFT data images of the aligned fiber matrices result in an output image where pixels are distributed in an elliptical shape since the pixel intensities are distributed to a specific orientation. Furthermore, the shape and height of the peaks, produced by the FFT analysis indicated the degree of fiber alignment. The narrower and higher peak indicates that the fibers are more highly aligned along with a specific axis. The orientation of random PDO fibers was disposed with various angles (Fig. 1E). Otherwise, the aligned PDO, PDO/Col and Lam-PDO/Col fibers showed the dominant distribution near 110°, 170° and 10°, respectively, whereas the random PDO fibers displayed a relatively broad distribution of fiber angle. Therefore, it was evidenced that the aligned fiber matrices were fabricated successfully by magnetic field-assisted electrospinning system.

Following a previous study, the alignment scaffold can induce the alignment of cells, which in turn, the alignment of cells can provide an effective guidance cue for the neurite outgrowth^61^. The diameters of random PDO, aligned PDO, aligned PDO/Col, and aligned Lam-PDO/Col core-shell fibers were found to be 1.25 ± 0.25, 1.85 ± 0.58, 1.39 ± 0.19, and 2.06 ± 0.87 μm, respectively (Figure S3B). The reason why the diameters of aligned fibers were thicker than those of random fibers could be the higher viscosity of their electrospinning solution. In addition, the fiber diameter of aligned PDO/Col matrices was more decreased than that of aligned PDO matrices because the effect of collagen blended in PDO^5^. The aligned Lam-PDO/Col core-shell matrices had the thickest fiber diameter because their electrospun conditions (coaxial electrospinning system) were different with uniaxial electrospinning. However, considering that the all matrices consisted micrometer-scale fibers, cell-matrix interactions could be effective because of their high surface area-to-volume ratio^11^.

### Physicochemical and mechanical properties of aligned Lam-PDO/Col core-shell matrices

**Figure 2:**
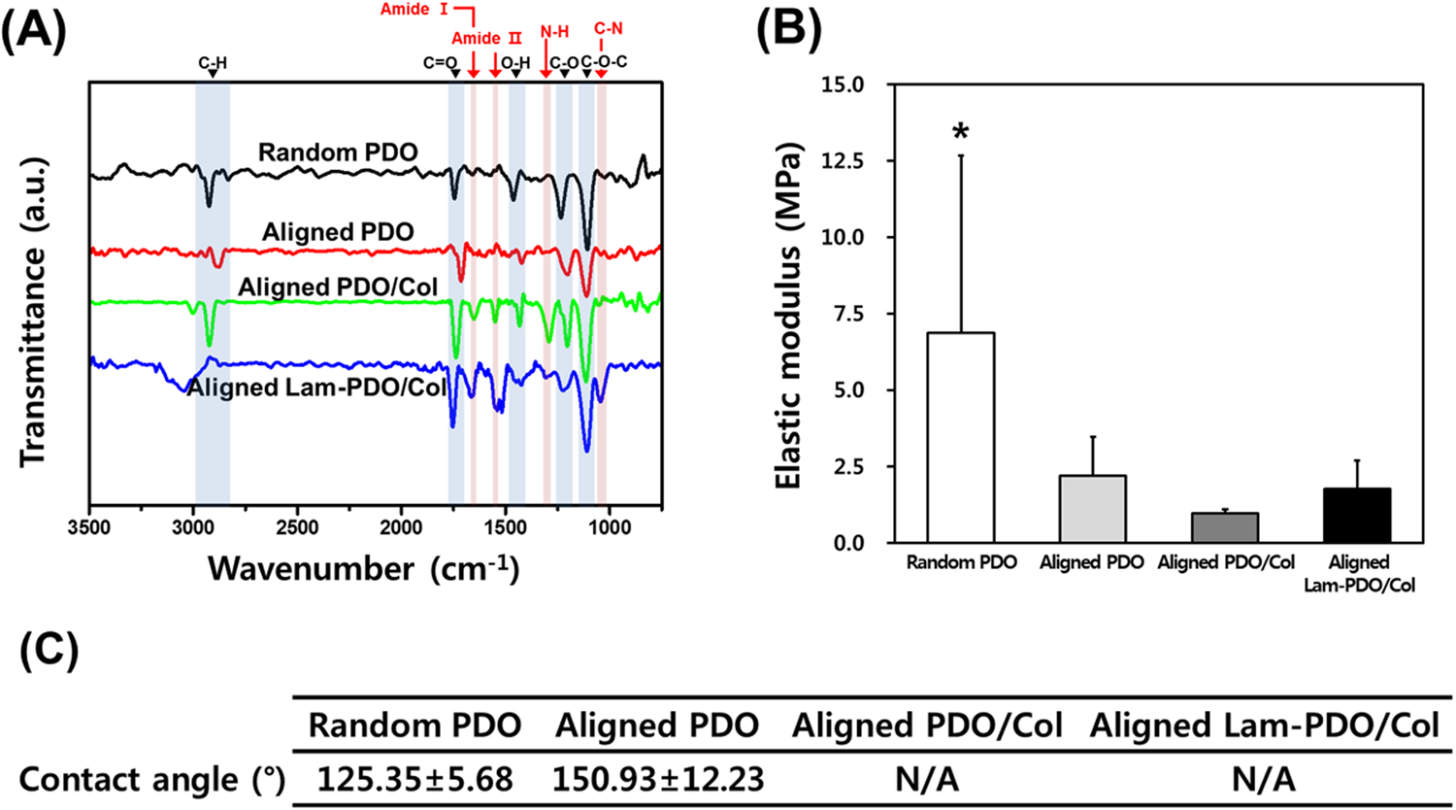
Physicochemical and mechanical characteristics of the random PDO, aligned PDO, aligned PDO/Col, and aligned Lam-PDO/Col core-shell matrices. (**A**) FTIR spectra of the random PDO, aligned PDO, aligned PDO/Col, and aligned Lam-PDO/Col core-shell matrices. (**B**) Elastic moduli of the random PDO, aligned PDO, aligned PDO/Col, and aligned Lam-PDO/Col core-shell matrices under a cross-head speed of 10 mm/min. An asterisk (*) denotes a significant difference compared with the other groups (*p* < 0.05). (**C**) Water contact angles of the random PDO, aligned PDO, aligned PDO/Col, and aligned Lam-PDO/Col core-shell matrices.

FTIR spectra were used in this work to analyze the compositions of matrices, and were presented in Fig. 2A. It was demonstrated that the FTIR spectrum of the aligned Lam-PDO/Col core-shell matrices included characteristic bands of PDO, Col and Lam. The C-H, C=O, O-H, C-O, and C-O-C bands were assigned to PDO, and the amide I, amide II and N-H band corresponded to Col and Lam^62,63^. In addition, the specific peak of Lam was also observed near 1035 cm^−1^, which represented the C-N stretching vibrations^64^. In the aligned Lam-PDO/Col core-shell spectrum, the C-N band outstands especially because not only collagen but also laminin have many C-N bonds in their chemical components. Consequently, the aligned Lam-PDO/Col core-shell matrices were fabricated efficiently, and the Col and Lam were incorporated successfully in the fibers.

The elastic moduli of the random PDO, aligned PDO, aligned PDO/Col, and aligned Lam-PDO/Col core-shell matrices were shown in Fig. 2B. The random PDO matrices exhibited significantly higher elastic modulus than aligned PDO matrices. This could be due to the fact that the entangled fibers in the random PDO matrices can make crossover points, which results in the formation of strong three-dimensional network structures, subsequently allows tensile load to be transferred between entangled fibers^65,66^. The elastic modulus of aligned PDO/Col matrices was more significantly decreased than that of aligned PDO because Col in the aligned PDO/Col matrices has poor mechanical properties^67^. The aligned Lam-PDO/Col core-shell matrices had the slightly higher elastic modulus than the aligned PDO/Col matrices, since the aligned Lam-PDO/Col core-shell matrices were less aligned than the aligned PDO/Col matrices. Meanwhile, because the aligned Lam-PDO/Col core-shell matrices were composed to liquid core including laminin, they had the most weakness of elongation at break (Figure S4 and Table S1). However, the tensile strength of the aligned matrices closely matches with the mechanical properties of acellularized rat peripheral nerves, suggesting that the aligned Lam-PDO/Col core-shell matrices could be appropriately used as nerve tissue engineering scaffolds^68^.

On the other hand, the interactions between cells and substrates are strongly dependent on the surface hydrophilicity of the substrates^69^. The water contact angles were 125.35 ± 5.68° for the random PDO matrices and 150.93 ± 12.23° for the aligned PDO matrices (Fig. 2C). The surfaces of random and aligned PDO matrices were hydrophobic. In addition, the water contact angle of the aligned matrices was slightly increased by fiber alignment because the aligned matrices had smaller pore size on their surface than random matrices^65^. On the other hand, the aligned PDO/Col and the aligned Lam-PDO/Col core-shell matrices perfectly absorbed the dropped water as soon as the water was dropped on the matrices (Figure S5, Movie S1 and S2). It was indicated that the aligned PDO/Col and Lam-PDO/Col core-shell matrices have a highly hydrophilic surface due to the intrinsic hydrophilic properties of Col and Lam^70^. These improvements in the surface hydrophilicity of matrices can enhance the cellular behaviors, such as initial cell attachment, proliferation and differentiation, as well as the interactions between cells and matrices^71^. Therefore, the aligned Lam-PDO/Col core-shell matrices are able to provide a suitable microenvironment for cell growth.

### *In vitro* laminin release and degradation profiles of aligned Lam-PDO/Col core-shell matrices

Lam was released in a sustained manner for up to 28 days (Figure S6A). A slight release during the earlier time within 7 days was observed, which was followed by a sustained release. After 28 days, the amount of Lam released from aligned Lam-PDO/Col core-shell matrices was detected constantly, reaching about 40%. This release profile is the characteristic of diffusion-controlled systems^6,72^. These results implied that the Lam was sufficiently incorporated into the core of aligned Lam-PDO/Col core-shell fibers, and the Lam was released continuously from the matrices in aqueous conditions. *In vitro* degradation of random PDO, aligned PDO, aligned PDO/Col, and aligned Lam-PDO/Col core-shell matrices was investigated by measuring the weight loss of each matrix. Figure S6B shows that the all matrices began a process of decomposition after 1 week and the mass of matrices gradually decreased until 4 weeks. The weight of random PDO and aligned PDO matrices slowly and gradually decreases for 4 weeks because PDO has long-term biodegradable time about 6 months^29,31^. However, the aligned PDO/Col and aligned Lam-PDO/Col core-shell matrices were fast degraded for initial 1 week, after that, their degradation behaviors tended to similar with those of random PDO and aligned PDO matrices. This result revealed that the majority of Col-blended shell fibers was degraded for initial 1 week, and the rest of fiber, PDO, were slowly disassembled for 4 weeks. On the other hand, since Lam in core of aligned Lam-PDO/Col core-shell fibers was steadily release, the weight loss of the aligned Lam-PDO/Col core-shell matrices was increased faster than other matrices. Therefore, the main release mechanism might be controlled by PDO/Col degradation and Lam diffusion from core of the fibers. In addition, the blended Col led to the increase of surface hydrophilicity, which facilitates the diffusion of water, followed by the enhancement of hydrolysis of aligned Lam-PDO/Col core-shell matrices. However, considering that the cumulative weight loss of aligned Lam-PDO/Col core-shell matrices during 4 weeks was approximately 13.8%, the matrices could maintain suitable structural integrity to support cell growth, indicating that the aligned Lam-PDO/Col core-shell matrices are able to provide a stable environment for cell growth.

### Cellular behaviors of HT-22 hippocampal neuronal cells on aligned Lam-PDO/Col core-shell matrices

**Figure 3:**
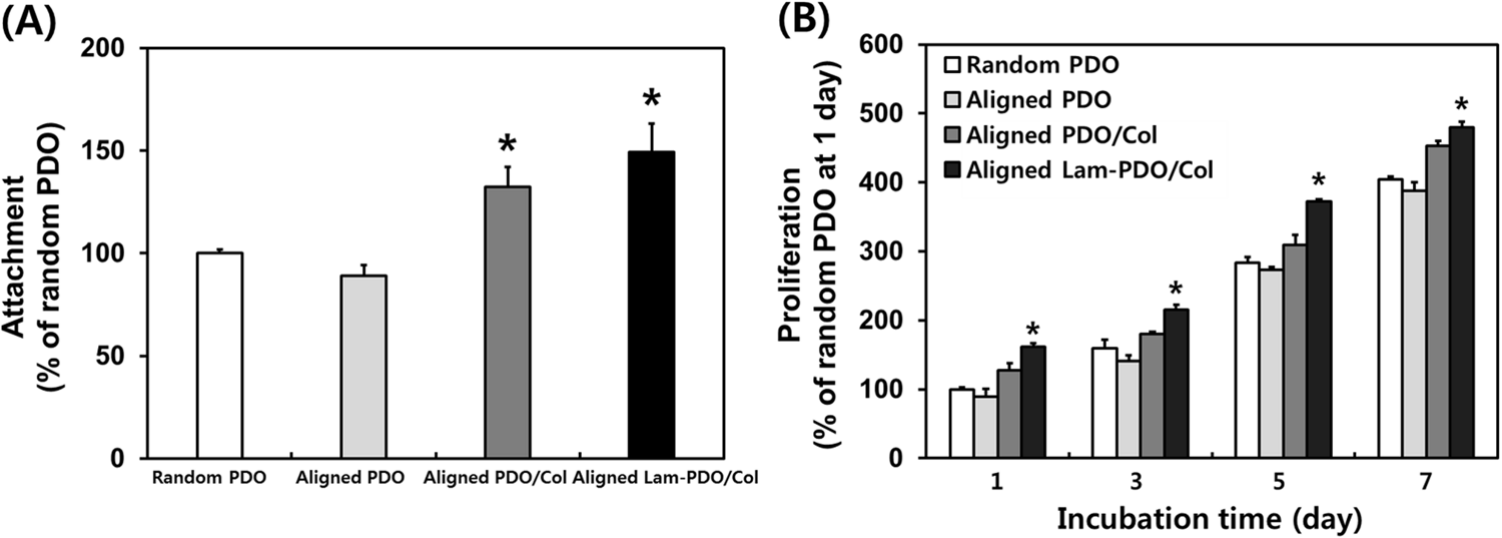
Cellular behaviors of HT-22 mouse hippocampal neuronal cells on the random PDO, aligned PDO, aligned PDO/Col, and aligned Lam-PDO/Col core-shell matrices. (**A**) Initial attachment and (**B**) proliferation of HT-22 mouse hippocampal neuronal cells on the random PDO, aligned PDO, aligned PDO/Col, and aligned Lam-PDO/Col core-shell matrices. An asterisk (*) denotes a significant difference compared with the random PDO matrices (*p* < 0.05). The data are presented as the average ± standard deviation of at least three independent experiments, each performed in duplicate on different cultures.

HT-22 hippocampal neuronal cells were cultured on the random PDO, aligned PDO, aligned PDO/Col, and aligned Lam-PDO/Col core-shell matrices to evaluate the initial cell attachment and proliferation. The initial cell attachments on the aligned PDO/Col and aligned Lam-PDO/Col core-shell matrices were significantly (*p* < 0.05) higher than those on the random PDO and aligned PDO matrices (Fig. 3A). This result well corresponds with those found in the previous studies where the surface hydrophilicity of the matrices strongly affects the initial cell attachment^67,69,71^. On the other hand, Fig. 3B indicated that the cells on all matrices steadily proliferated during the culture period (7 days). However, it was remarkable that the cell proliferation on the aligned Lam-PDO/Col core-shell matrices was significantly (*p* < 0.05) enhanced as compared with those on the other matrices. This could be because of the synergistic effects of the ameliorated the surface hydrophilicity of aligned Lam-PDO/Col core-shell matrices and the ability of Col and Lam to promote cell proliferation^67,69,71,73^. It has been commonly acknowledged that Col has an outstanding biocompatibility and cell affinity, leads to promoted cell adhesion and proliferation^74^. Moreover, the previous studies demonstrated that Lam can promote neurite function by stimulating cell mitosis, and can enhance the migration and proliferation of nerve cells^75^. In addition, Lam has a specific peptide sequence (Ile-Lys-Val-Ala-Val), found on the C-terminus of the α1 chain of Lam, and it has been found to be capable of promoting neuronal cell adhesion, proliferation and neurite outgrowth^75^. Therefore, both Col and Lam enable to synergistically enhance HT-22 cell attachment and proliferation, suggesting that the aligned Lam-PDO/Col matrices are able to effectively enhance the initial cell attachment and proliferation by the synergistic effects of Col and Lam.

**Figure 4:**
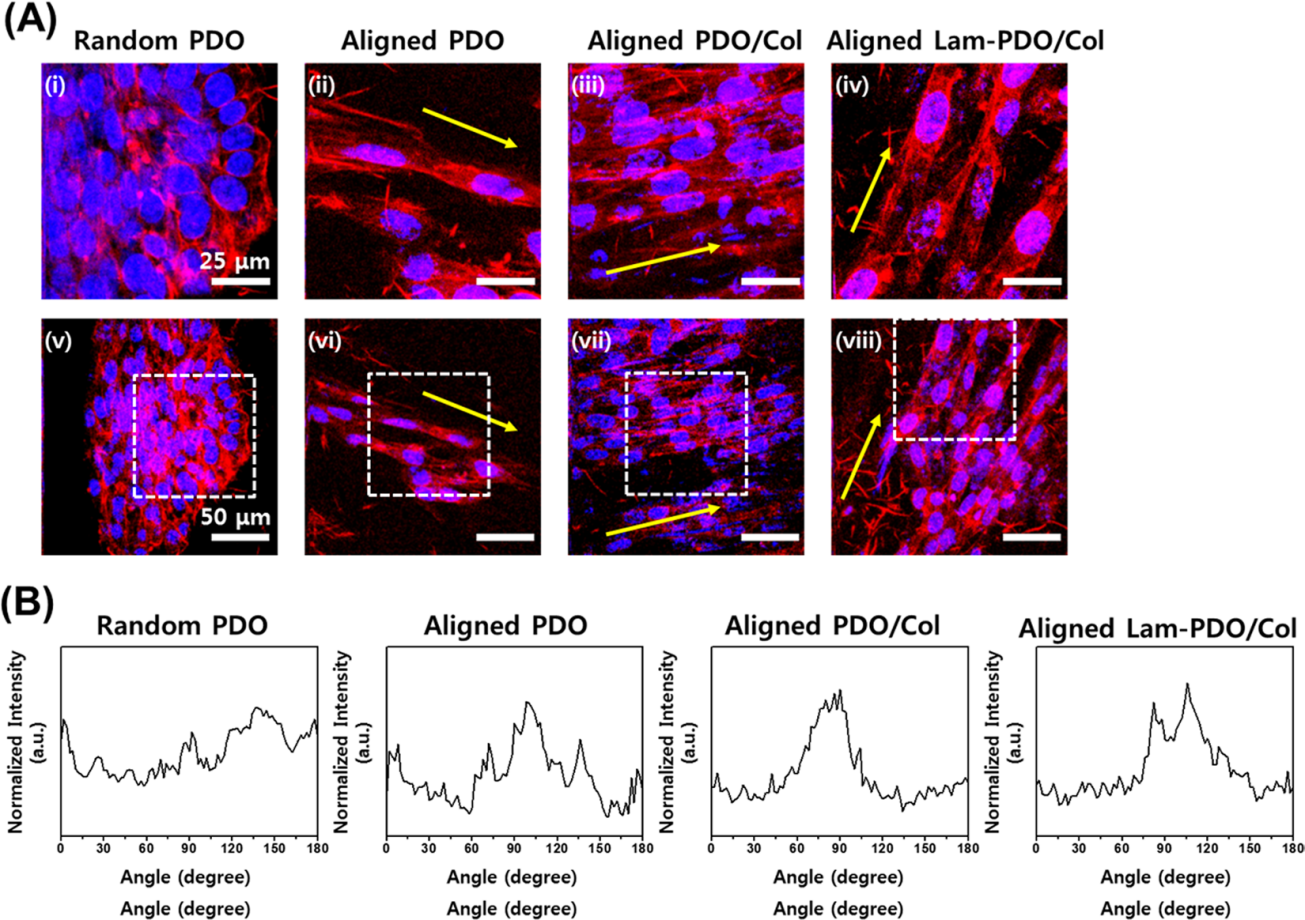
Cell morphologies and alignment of HT-22 mouse hippocampal neuronal cells on the random PDO, aligned PDO, aligned PDO/Col, and aligned Lam-PDO/Col core-shell matrices. (**A**) Two-photon excitation fluorescence images of HT-22 mouse hippocampal neuronal cells on the random PDO, aligned PDO, aligned PDO/Col, and aligned Lam-PDO/Col core-shell matrices at 5 days of culture. (i-iv) Magnified images of the region enclosed by the dashed white square in (v–viii). The cell nuclei were counterstained with DAPI (blue), and the F-actins were stained with TRITC-labelled phalloidin (red). Yellow arrows indicate the direction of aligned fibers. All photographs shown in this figure are representative of six independent experiments with similar results. (**B**) Quantitative analysis of the cell alignment on the random PDO, aligned PDO, aligned PDO/Col, and aligned Lam-PDO/Col core-shell matrices. Note the distinctive pixel intensity created by the FFT output image containing aligned HT-22 cell information.

To identify the morphologies of HT-22 hippocampal neuronal cells on the random PDO, aligned PDO, aligned PDO/Col, and aligned Lam-PDO/Col core-shell matrices, two-photon excitation microscope images were obtained after 5 days of cell culture on the matrices (Fig. 4A). The cells on the random PDO matrices showed aggregated morphology with poor developed F-actins. On aligned PDO matrices, the F-actins were aligned along with the direction of fibers, although the number of cells was significantly lower than other groups. However, on the aligned PDO/Col and aligned Lam-PDO/Col matrices, HT-22 hippocampal neuronal cells were favorably grown and widely spread out on the matrix surface. In addition, the F-actins of cells were developed parallel to the aligned fiber direction (yellow arrows). These results indicated that the aligned fibers incorporated Col and Lam could improve cell proliferation. Moreover, the aligned PDO/Col and Lam-PDO/Col core-shell matrices could induce the alignment of neuronal cells by providing a suitable topographical guidance cue. The alignments of F-actins were analyzed by FFT methods to quantify the cell alignments (Fig. 4B). The cells on the random PDO matrices were randomly oriented, and the angles between F-actins and aligned fiber direction showed a relatively broad distribution. In contrast, cells on aligned PDO, PDO/Col and Lam-PDO/Col matrices were aligned parallel to the direction of aligned fibers, and the angles were dominant at a specific degree. These results indicated that the Lam-PDO/Col core-shell matrices can not only promote cellular behaviors, but can also effectively induce cell alignment. In previous studies, it has been reported that the cell elongation and alignment increase the efficiency of neuronal differentiation^76^. Hence, the neurogenic differentiation of HT-22 hippocampal neuronal cells on the matrices was investigated.

**Figure 5:**
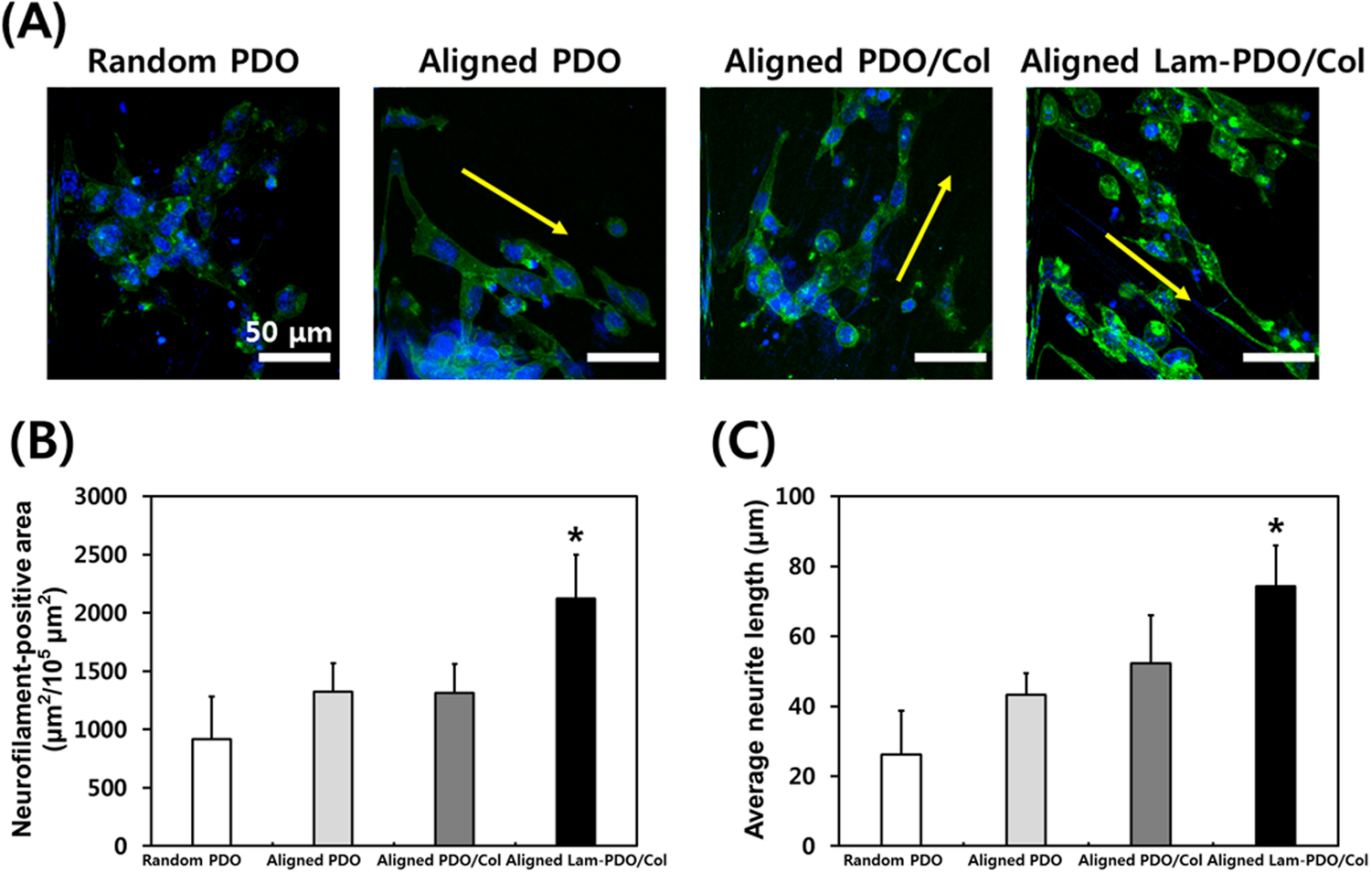
Immunofluorescence staining analysis of neurite outgrowth and HT-22 mouse hippocampal neuronal cell alignment on the random PDO, aligned PDO, aligned PDO/Col, and aligned Lam-PDO/Col core-shell matrices. (**A**) Two-photon excitation fluorescence images of neurite outgrowth. The HT-22 mouse hippocampal neuronal cells were cultured in growth media for 5 days, and further cultured in differentiation media for 7 days on the random PDO, aligned PDO, aligned PDO/Col, and aligned Lam-PDO/Col core-shell matrices. The cell nuclei were counterstained with DAPI (blue), and the neurofilaments were immunofluorescence stained with FITC-labelled antibody for the detection of neurofilaments (green). Yellow arrows indicate the direction of aligned fibers. All photographs shown in this figure are representative of six independent experiments with similar results. (**B**) Quantification of neurofilament-positive area and (**C**) average neurite outgrowth of HT-22 mouse hippocampal neuronal cell. An asterisk (*) denotes a significant difference compared with the other groups (*p* < 0.05). The data are presented as the average ± standard deviation of at least three independent experiments, each performed in duplicate on different cultures.

The HT-22 hippocampal neuronal cells on the matrices were incubated in differentiation medium to induce neurite outgrowth, which is evidence of neuritogenesis, and the differences in neurite outgrowth were shown in Fig. 5A. On the random PDO matrices, the neurites of cells appeared less spread-out than on the aligned matrices. Although neurites of the cells on the aligned PDO matrices were more elongated than that on the random PDO matrices, they showed just short neurite outgrowth and their population was less than other matrices. On the other hand, the neurofilament morphologies of cells on aligned PDO/Col and aligned Lam-PDO/Col core-shell matrices had spindle edge shapes, and were aligned along the aligned fibers. In addition, the numbers of cells on aligned PDO/Col and aligned Lam-PDO/Col core-shell matrices were more than the random and aligned PDO matrices. It was demonstrated that the effect of contact guidance provided by the aligned fibers appeared to be more dramatic than the randomly oriented fibers. Previous studies revealed that the aligned nanofiber matrices can directly induce cell alignment, which allows guided and promoted neurite outgrowth^76–78^. Interestingly, our results also showed that the contact guidance provided by the aligned fibers could improve the neurite outgrowth. The rational design of scaffolds is crucial for controlling neurite outgrowth and peripheral nerve regeneration. In particular, the aligned fiber structure can effectively facilitate neurite outgrowth by providing anisotropic cues, which can result in the faster and better peripheral nerve regeneration^79–81^. In the present study, when comparing neurite outgrowth on random and aligned PDO matrices, cells on aligned PDO matrices showed better neurite outgrowth than random PDO matrices. In addition to aligned PDO matrices, other aligned matrices (i.e. aligned PDO/Col and Lam-PDO/Col matrices) can also provide anisotropic cues for facilitating neurite outgrowth. Hence, the specific structural feature of aligned Lam-PDO/Col matrices is highly desirable for neural tissue regeneration as well as nerite outgrowth. Meanwhile, blended Col in aligned PDO/Col and Lam-PDO/Col matrices played an important role in promoting cell attachment and proliferation. It has been widely acknowledged that Col blended fiber has an effect on the better cell viability for differentiation than other matrices without Col^76^. HT-22 cells on aligned Lam-PDO/Col matrices were clearly well grown, and presented long neurite extensions. The neurofilament-positive area (μm^2^/10^5^ μm^2^) and neurofilament length were measured to quantitatively compare the nerite outgrowth of HT-22 hippocampal neuronal cells on the matrices (Fig. 5B and C). The HT-22 hippocampal neuronal cells on the aligned Lam-PDO/Col core-shell matrices showed remarkably high neurofilament-positive area. Moreover, average neurite length of HT-22 cells on the aligned Lam-PDO/Col core-shell matrices was also significantly (*p* < 0.05) increased. The neurite outgrowth of the cells on aligned Lam-PDO/Col core-shell matrices was longest among the cells on other matrices. These results correspond well with those found in the earlier studies. Lam is the most popular promoting factor for the growth and differentiation of neuronal cells^34,75^. Lam plays a pivotal role in neuronal cell growth and axonal extension, and is actively synthesized and secreted by astrocytes when nerve injury is caused^82–84^. Therefore, polymer fiber matrices modified with Lam have been employed for promoting neuronal cell growth and neurite out growth. It has been found that the Lam-incorporated polymer fibers can not only guide neurite extension, but can also promote peripheral nerve regeneration^34,85^. However, the stimulatory effects of Lam are highly dependent on the modification techniques, such as covalent immobilization, physical adsorption or blending. In addition, the Lam-blended or Lam-coated polymer fibers typically show an initial burst release, which is not desirable for nerve tissue regeneration, because Lam cannot achieve sufficient effects during the entire tissue regeneration process. On the other hand, Lam in the core of aligned Lam-PDO/Col core-shell fibers was steadily and continuously released from fibers (Figure S6A), which could directly and sustainedly affect the neuronal cell growth and neurite outgrowth during sufficient time (more than 4 weeks) to allow nerve tissues to be regenerated. Therefore, apart from the aligned structure of Lam-PDO/Col matrices, Lam encapsulated in the Lam-PDO/Col core-shell fibers is the critical key to promote neuritogenesis as well as rapid neurite outgrowth. Hence, our results indicated that the aligned Lam-PDO/Col core-shell matrices can effectively induce cell alignment and promote neuritogenesis, as well as facilitating cellular behaviors.

## Conclusions

In this study, we have established a novel approach to producing aligned matrices with core-shell structure by modifying the electrospinning process. The main contributions of this study can be summarized as follows: (1) We successfully fabricated the aligned Lam-PDO/Col core-shell fibers, which can provide both topographical and biochemical cues for promoting neuritogenesis, by magnetic field-assisted electrospinning with the coaxial system. (2) The aligned Lam-PDO/Col core-shell fiber matrices have desirable structural, physicochemical and mechanical properties for use as tissue engineering scaffolds. (3) The aligned fiber structure can effectively facilitate neurite outgrowth by providing anisotropic cues, while the Lam released from the fibers in a sustained manner is the critical key to promote neuritogenesis as well as rapid neurite outgrowth. (4) The aligned Lam-PDO/Col core-shell fiber matrices can effectively promote the hippocampal neuronal cell growth, and favorably enhance their neurogenic differentiation and neurite outgrowth. Our results revealed that the aligned Lam-PDO/Col core-shell fiber matrices are one of the most promising approaches for facilitating neuritogenesis and promoting neural tissue regeneration. In conclusion, it is suggested that the aligned Lam-PDO/Col core-shell fiber matrices can serve as potential scaffolds for neuritogenesis and neural tissue engineering.

## Electronic supplementary material

Supporting Information

Supplementary Movie S1

Supplementary Movie S2
