# Supplementary material for: Aligned laminin core-polydioxanone/collagen shell fiber matrices effective for neuritogenesis
Source: Sci Rep. 2018 Apr 3;8:5570. doi: 10.1038/s41598-018-23958-3 (PMC5882927; doi:10.1038/s41598-018-23958-3)
Supplement: Supplementary file 1 — Supporting Information [file 41598_2018_23958_MOESM1_ESM.doc]

Supporting Information

Aligned laminin core-polydioxanone/collagen shell fiber matrices effective for neuritogenesis

Su-Jin Song^1,†^, Yong Cheol Shin^2,†^, Sung Eun Kim^1^, Il Keun Kwon^3^, Jong-Ho Lee^4^, Suong-Hyu Hyon^5^, Dong-Wook Han^1,*^ & Bongju Kim^6,**^

^1^Department of Cogno-Mechatronics Engineering, College of Nanoscience & Nanotechnology, Pusan National University, Busan 46241, Republic of Korea; ^2^Research Center for Energy Convergence Technology, Pusan National University, Busan 46241, Republic of Korea; ^3^Department of Dental Materials, School of Dentistry, Kyung Hee University, Seoul 02447, Republic of Korea; ^4^Department of Oral and Maxillofacial Surgery, School of Dentistry, Seoul National University, Seoul 03080, Republic of Korea; ^5^Center for Fiber and Textile Science, Kyoto Institute of Technology, Matsugasaki, Kyoto 606-8585, Japan; ^6^Dental Life Science Research Institute, Seoul National University Dental Hospital, Seoul 03080, Republic of Korea

^†^S.-J.S. and Y.C.S contributed equally to this work.

Correspondence and requests for materials should be addressed to D.-W.H. (email: nanohan@pusan.ac.kr) and B.K. (email: bjkim016@gmail.com)


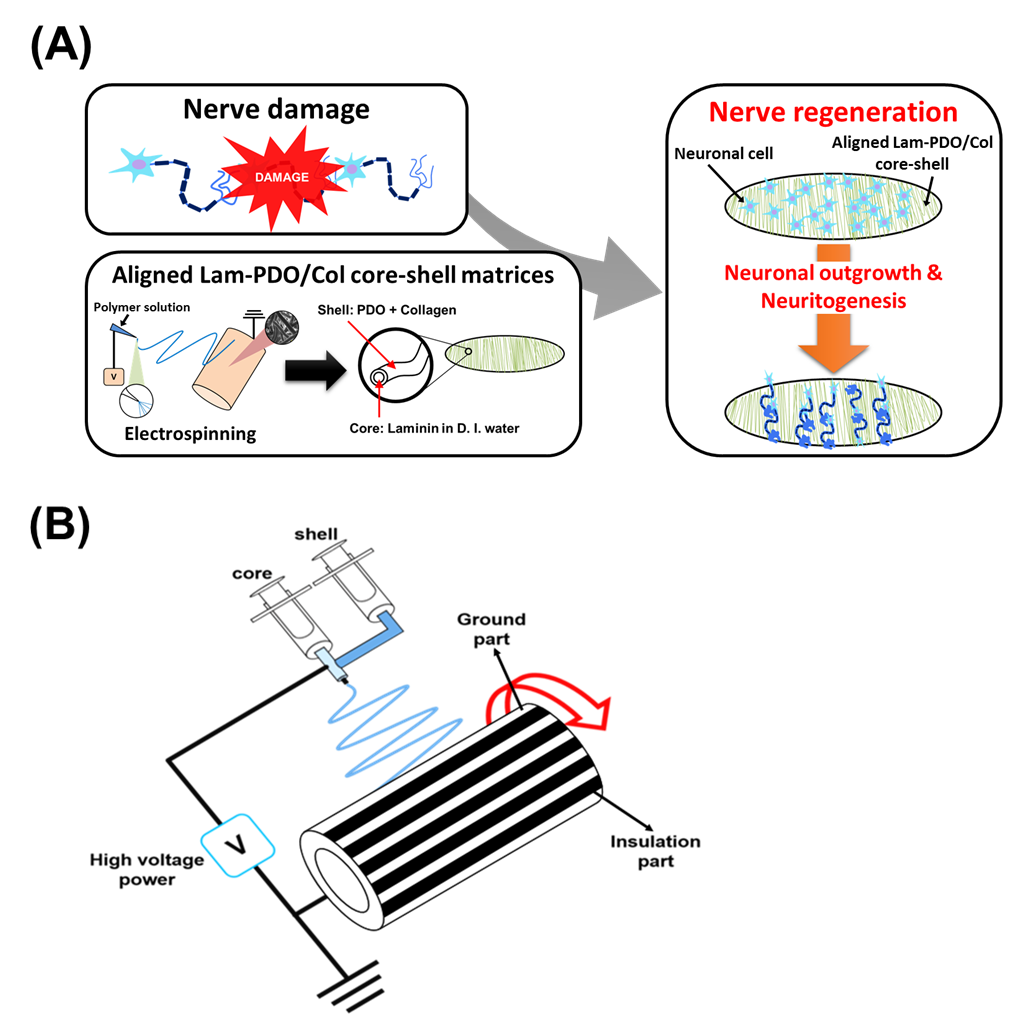


**Supplementary Figure S1.** (A) Schematic overview of the aligned laminin core-polydioxanone/collagen shell (Lam-PDO/Col) fiber matrices effective for neuritogenesis. (B) Schematic diagram of the magnetic field-assisted electrospinning with the coaxial system for the fabrication of the aligned Lam-PDO/Col core-shell fiber matrices.


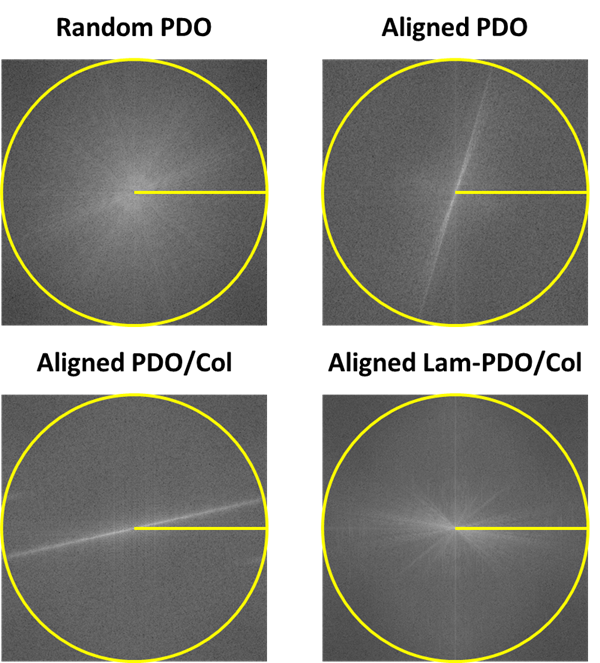


**Supplementary Figure S2.** FFT output images of the random PDO, aligned PDO, aligned PDO/Col, and aligned Lam-PDO/Col core-shell matrices. Yellow circle is radical projection.


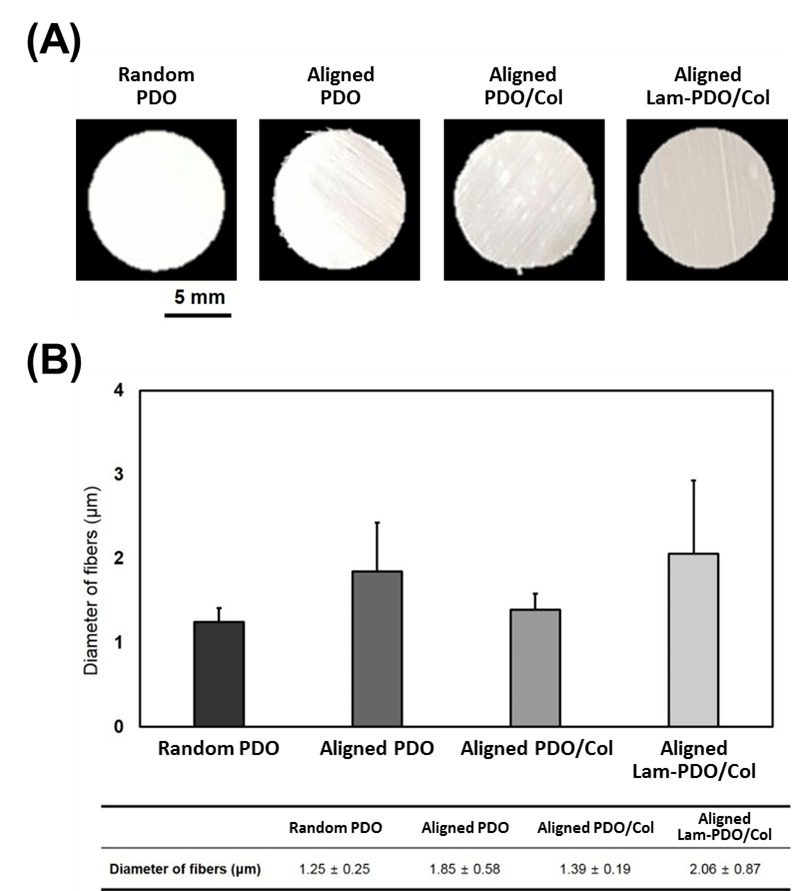


**Supplementary Figure S3.** (A) Digital photographs of the random PDO, aligned PDO, aligned PDO/Col, and aligned Lam-PDO/Col core-shell matrices. All photographs shown in this figure are representative of six samples with similar results. (B) Average diameters of the random PDO, aligned PDO, aligned PDO/Col, and aligned Lam-PDO/Col core-shell matrices.


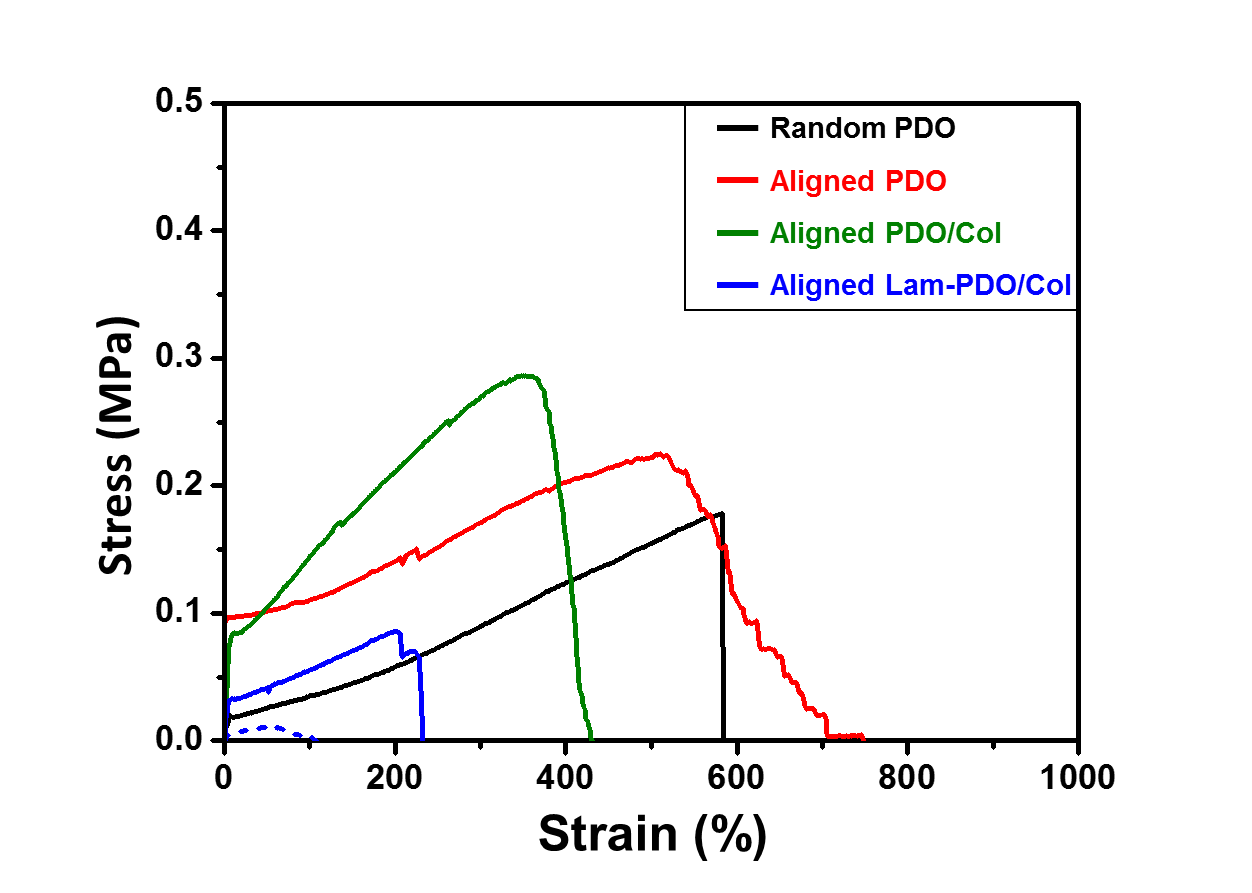


**Supplementary Figure S4.** Stress–strain curves of the random PDO, aligned PDO, aligned PDO/Col, and aligned Lam-PDO/Col core-shell matrices under a cross-head speed of 10 mm/min. The aligned Lam-PDO/Col core-shell matrices were analyzed in longitudinal (blue solid line) and transverse (blue dotted line) directions.


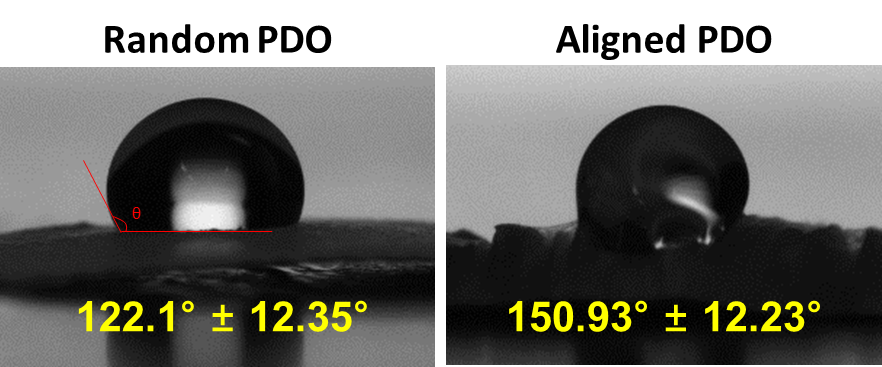


**Supplementary Figure S5.** Water contact angle images for water droplets on the random PDO and aligned PDO matrices. Water contact angles of the matrices were measured by the sessile drop method.


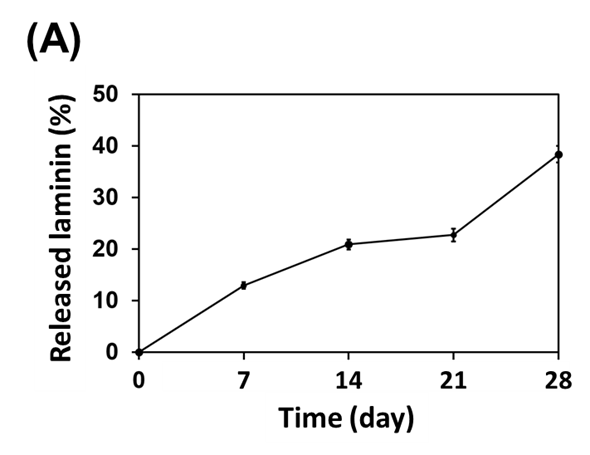


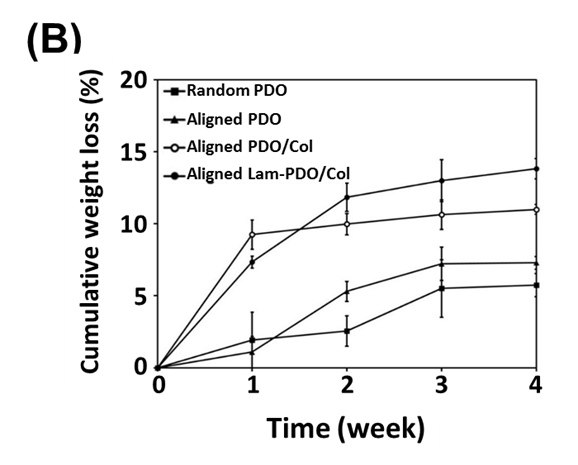


**Supplementary Figure S6.** (A) *In vitro* laminin release profile from the aligned Lam-PDO/Col core-shell matrices. The released laminin is plotted as the percentage of its loaded amounts. (B) *In vitro* degradation analysis of the random PDO, aligned PDO, aligned PDO/Col, and aligned Lam-PDO/Col core-shell matrices. The cumulative weight loss of the matrices was determined as the percentage ratio of the dry weight of each matrices at each time to its initial weight.

**Supplementary Table S1.** Mechanical properties (tensile strength, elastic modulus and elongation at break) of the random PDO, aligned PDO, aligned PDO/Col, and aligned Lam-PDO/Col core-shell matrices.


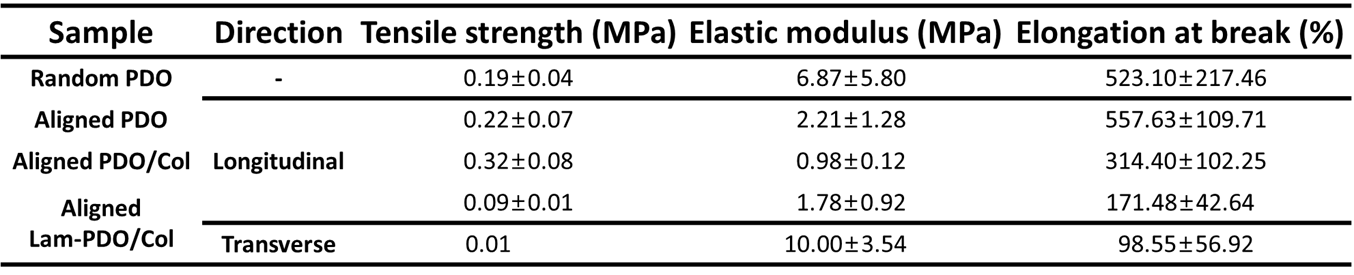


**Captions for supporting Movies**

**Supplementary Movie S1:** This movie shows the water contact angle measurement video for the aligned PDO/Col matrices. Water droplets were perfectly absorbed as soon as the water was dropped on the aligned PDO/Col matrices.

**Supplementary Movie S2:** This movie shows the water contact angle measurement video for the aligned Lam-PDO/Col core-shell matrices. Water droplets were perfectly absorbed as soon as the water was dropped on the aligned Lam-PDO/Col core-shell matrices.
